# Prediction and Potential Preventions for the Development of Posttraumatic Osteoarthritis after the Terrible Triad Injury: A Multicenter Risk Factors Study

**DOI:** 10.1155/2020/6508781

**Published:** 2020-06-21

**Authors:** Jingjing Li, Di Lu, Wenxiao Lin, Qinglong Li, Jing Hu, Ding Xu, Youming Zhao, Weijun Guo

**Affiliations:** ^1^Department of Radiology, The Second Affiliated Hospital and Yuying Children's Hospital of Wenzhou Medical University, Wenzhou, China; ^2^Department of Orthopedics, The Second Affiliated Hospital and Yuying Children's Hospital of Wenzhou Medical University, Wenzhou, China; ^3^Department of Orthopedics, Sichuan Orthopedics Hospital, Sichuan, China; ^4^Department of Orthopedics, Wenzhou People's Hospital, Wenzhou, China; ^5^Department of Orthopedics, Shangyu People's Hospital, Shaoxing, China

## Abstract

**Objective:**

A multicenter study to evaluate risk factors for the development of moderate or severe posttraumatic osteoarthritis (PTOA) and to find potential preventions.

**Methods:**

We conducted a retrospective multicenter study including the terrible triad injury (TTI) patients with surgical treatment from January 2007 to November 2014. Demographics, injury information, and treatment history were obtained retrospectively. According to the Broberg and Morrey criterion, 198 included patients were sorted into two groups: the mild or no PTOA and moderate or severe PTOA. Uni- and multivariate logistic regression analyses were used to identify risk factors for moderate or severe PTOA.

**Results:**

Moderate or severe PTOA was present in 64 patients (32.3%). Significant risk factors were Mason III radial head fracture (OR 4.049, 95% CI 1.877-8.736, *p* < 0.001), medial collateral ligament injury (OR 5.120, 95% CI 1.261-20.790, *p* = 0.022), and heavy use of elbow (OR 2.333, 95% CI 1.060-5.136, *p* = 0.035). Besides, patients suffered subluxation (*p* = 0.007) and those with more risk factors had a higher risk to develop moderate or severe PTOA.

**Conclusions:**

Moderate or severe PTOA was common after the TTI. Patients need to be counseled about avoiding heavy use of the elbow, especially for those with Mason III radial head fractures. Surgeons should be aware of the recurrent instability of the elbow.

## 1. Introduction

Elbow dislocation associated with both radial head and coronoid fractures earned its eponym “terrible triad injury” (TTI) for decades because of the poor prognosis [1]. With the improvement of knowledge on elbow biomechanics and pathoanatomy, the current standard strategy appeared to have yielded more favorable outcomes [2, 3]. However, challenges persist. Complications like joint stiffness, posttraumatic osteoarthritis (PTOA), heterotopic ossification, and ulnar nerve symptoms continue to affect the prognosis of the TTI [4, 5].

PTOA is difficult to manage, especially in the high-demand or active population. It presents a challenge for both patients and orthopedic surgeons. The correlation between radiographic evidence of PTOA and symptoms is limited, especially in mild ones [9, 10]. When PTOA has aggravated, several treatments would be considered for moderate or severe PTOA. Unfortunately, cartilage damage during these stages cannot be reversed [6]. Pharmacologic therapies (including anti-inflammatory agents, joint injection with corticosteroid or hyaluronic acid) offered only relief of pain and inflammation instead of any long term benefit [7]. Arthroplasty is recommended only for the older and low-demand patients [8], whereas TTI is most common in mid-age patients who are the main labor force and difficult to comply with long-term activity restrictions. Increased demands placed throughout their lifetime make arthroplasty only a remedy option [9]. Hence, it is important to understand the risk factors for the development of moderate or severe PTOA to provide a theoretical basis for early prevention, while delaying elbow arthroplasty to the extent possible. But little is known.

Therefore, we designed a multicenter retrospective study of TTI patients after surgical treatment. We used uni- and multivariate logistic regression analyses to identify risk factors for the development of moderate or severe PTOA and to find potential preventions.

## 2. Material and Methods

### 2.1. Study Design and Patients

This study was approved by the Ethics Committee of our hospitals. We conducted a retrospective cohort study including consecutive patients operated upon at our four hospitals from January 2007 to November 2014 for the TTI of the elbow treated by ORIF. Inclusion criteria were (1) TTI patients with surgical treatment, (2) an age of 18 years or older, and (3) medical information with elbow radiograph of more than 3 years. Patients with pathological fractures, prior elbow trauma or osteoarthritis, or incomplete medical information were excluded, as well as those treated with arthroplasty or resection for radial head fractures.

Operations were performed by different surgeons based on the standard strategy [10]. In brief, coronoid fractures were treated with cannulated screws or miniscrews. Otherwise, the anterior capsular repair was performed for Regan-Morrey type I and some type II fractures. Then, radial head fractures of Mason II and III were treated with cannulated screws or miniscrews, and small fragments were fixed by K wires. Plates were used for radial neck fractures. Mason I fractures were treated conservatively. All lateral collateral ligaments were repaired as well as the extensor origin and the posterolateral capsule. Afterward, elbow stability was evaluated. Medial collateral ligament (MCL) repair or hinged external fixator was performed according to the extent of valgus stability.

### 2.2. Data Collection and Potential Risk Factors

Demographics, injury information, and treatment history were extracted from the medical records. We also contacted some patients to confirm details like occupation after fracture healing. All these data were used to evaluate the risk factors as follows:

Included demographics were age; gender; dominant arm (yes/no); hypertension (yes/no), defined as “systolic BP ≥140 mmHg, and/or diastolic BP ≥90 mmHg, and/or use of antihypertensive medicine within 2 weeks” [11]; diabetes mellitus (yes/no), defined as “a single raised glucose reading with symptoms or history of mellitus on medication or with the documented complications” [12]; former or current smokers who had smoked >100 cigarettes or reported regular cigar or cigarillo smoking were categorized as smoking (yes/no) [13]; alcohol abuse (yes/no), defined as alcoholic drinks ≥25 g per day in the past 12 months [14]; and patients with BMI ≥ 25 were grouped as overweight (yes/no).

Injury information was fracture types of both radial head and ulna coronoid process. To measure the extent of medial instability, a valgus stress test was performed under fluoroscopy before surgery. Patients with joint space widening more than 3 mm were defined as MCL injury.

Surgical approach (lateral, posterior, and lateral with medial) and the time from initial injury to surgery were obtained through the treatment records. Occupations with a history of heavy use of upper extremities, which have been suggested in the previous study, were sorted into “heavy use” (yes/no), including manual laborer, weight lifter, farmers, health care, and child care [15].

### 2.3. Radiographic Assessment

Radiographic assessment was performed by two investigators (including one radiologist and one orthopedic surgeon). A consensus was made after a review by a senior surgeon if there were any disagreements. Preoperative radiographs were reviewed to identify the fracture types. Coronoid fractures were categorized according to the Regan and Morrey classification [16], and radial head fractures were classified based on the original Mason classification [17]. Since PTOA was reported more commonly in Mason III fractures [18], we divided radial head fractures type into groups I/II and III.

Postoperative radiographs were evaluated after surgeries and at the final follow-up after surgery. Subluxation was defined as incongruity of the ulnohumeral joint. PTOA was defined as the presence of radiographic change (joint space narrowing with/or osteophyte formation), and their severity was classified into four grades according to the Broberg and Morrey criteria [19]: grade 0 (without radiographic arthritis), grade 1 (slight joint space narrowing with/or minimum osteophyte formation), grade 2 (moderate joint space narrowing with/or moderate osteophyte formation), and grade 3 (severe degenerative change and joint destruction). All patients were sorted into the mild or no arthritis (grade 0 or 1) and moderate or severe arthritis (grade 2 or 3) groups.

### 2.4. Statistical Analyses

Group distributions were analyzed using an independent-sample *t*-test, and proportions among groups were compared by chi-square and Fisher's exact test. To identify potential predictors, we used univariate logistic analysis to access the association between demographics, injury information, treatment history, and the development of moderate or severe PTOA. The risk factors with a *p* value < 0.05 in the univariate analysis were selected for the multivariate logistic regression analysis. Hosmer-Lemeshow test was used to appraise the regression model fit to the data. Odds ratios (ORs) were presented with 95% confidence intervals (CIs), and a *p* value < 0.05 was set for significance. The incidence of moderate or severe PTOA was analyzed according to the number of risk factors with each patient. Finally, Mantel-Haenszel test was performed to analyze the correlation between injury types. Statistical analyses were performed using SPSS version 24.0.

## 3. Results

We excluded 92 cases among 290 consecutive TTI patients, because of an incomplete medical record for a minimum of 3 years (*n* = 29), a history of previous trauma or osteoarthritis (*n* = 15), and treatment with arthroplasty (*n* = 48). Finally, 198 patients with a mean follow-up of 42.8 months (range, 36-63 months) were analyzed in this study. Among them, 64 (32.3%) patients had radiographic signs of moderate or severe PTOA with 60 classified as grade 2 and 4 as grade 3. While in another group, 33 patients were grade 1 and 101 were without PTOA (grade 0). The demographics, injury, and treatment characteristics were shown in Table 1.

**Figure 1:**
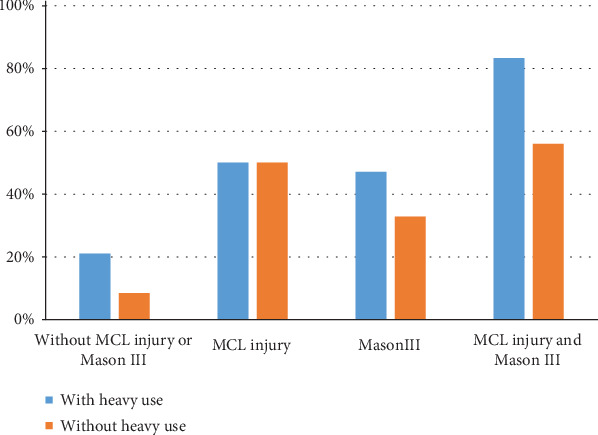
Incidence of moderate or severe PTOA according to presence or absence of heavy use of the elbow and an associated Mason III fracture and/or MCL injury.

Univariate analysis indicated that six risk factors among 14 variables were potential predictors, including alcohol abuse, occupation, coronoid and radial head fracture types, MCL injury, and surgical approach (Table 2). An incidence greater than 50% was found in patients with type III coronoid fracture (68.2%) and MCL injury (62.2%). After multivariate logistic analysis, occupation with heavy use of upper extremities (OR 2.333, 95% CI 1.060-5.136, *p* = 0.035), Mason III radial head fracture (OR 4.049, 95% CI 1.877-8.738, *p* < 0.001), and MCL injury (OR 5.120, 95% CI 1.261-20.790, *p* = 0.022) were independently associated with the development of moderate or severe PTOA and constituted the final model (Table 3). The Hosmer-Lemeshow test indicated a good fit (*p* = 0.840) on 77.8% occasions. The incidences according to these three factors are shown in Figure 1. Furthermore, the incidence of moderate or severe PTOA increased substantially from a low incidence (8.5%) in patients with no risk factors to a higher incidence (58.6%) in the presence of two or more risk factors (Table 4). In addition, severe coronoid fracture was correlated with MCL injury (*R* = 0.663, *p* < 0.001) and a more severe radial head fracture (*R* = 0.257, *p* < 0.001).

All patients had a concentric reduction of ulnohumeral joint after surgery. However, subluxation was found in sixteen patients (8.1%) within the first two weeks. Seven of them treated with a second surgery; the others were treated with external fixator or plaster. Besides, patients who suffered subluxation were more likely to develop moderate or severe PTOA (*p* = 0.007). Other complications included 17 cases of transient neuropathy (8.6%) which disappeared within 4 months (2 weeks to 4 months), 11 cases of delayed union of the fractures (5.6%), 4 cases of nonunion of the radial head that treated with resection, and 4 cases of local superficial infection (2%) that healed after using antibiotics. Six cases had a hardware migration (3%). The implant was removed in 4 of them, and the remaining 2 patients had a slight K-wire shift from the radial head without a secondary surgery. Forty-six patients had heterotopic ossification, of which 12 required a secondary surgery including heterotopic bone removal and elbow release. All these data were shown in Table 5.

## 4. Discussion

Moderate or severe elbow PTOA developed in 32.3% of patients on average 42.8 months after the terrible triad injury treated with ORIF. Occupation with heavy use of upper extremities, Mason III radial head fracture, and MCL injury significantly increased the risk of moderate or severe elbow PTOA as defined by the Broberg and Morrey grades 2-3. Besides, the presence of more risk factors was associated with a substantially increased incidence of moderate or severe elbow PTOA.

It is reported that Mason III radial head fracture is more likely to develop PTOA than Mason I or Mason II [18]. Complex radial fracture is difficult for surgical fixation. It increases the risk of poor reduction, which can lead to early PTOA [20]. However, some studies indicated that the displacement of the radial head was more related to the limitation of forearm rotation, not PTOA [21, 22]. Due to the different injury patterns between the TTI and isolated radial head fracture, it is still important to verify the relationship between PTOA and the severity of radial head fracture in the TTI. However, information is sparse. We found that moderate or severe PTOA tended to occur in the TTI patients with Mason III radial head fractures. It is known that increase stress on the articular surface can damage cartilage [20]. While compared with isolated radial head fractures, the TTI involves more structural damage of the elbow, including ligaments and coronoid process. Damage to these structures increases the valgus stress on the radial head [23, 24]. We also found that Mason III fractures were accompanied by more severe damage to the capsuloligamentous structures, reflecting higher energy damage to the elbow structures compared to Mason I or II fractures. Besides, in line with a previous study, we also found that Mason III fractures were more related to severe MCL injury [25]. It further increases the stress on the humeroradial joint which might be another reason.

It should be noted that we excluded patients treated with arthroplasty for radial fractures. Shore et al. [26] reported that 74% of patients developed PTOA after treating with metallic arthroplasty. And silicone implant was revealed to worsen arthritis secondary to elbow injury due to siliconitis [18]. We were unable to conduct a subgroup analysis for different treatments due to insufficient cases. It may not influence our results because arthroplasty was commonly used to treat comminuted radial head fractures in the TTI. And in a series of 39 patients with TTI, Watters et al. [27] reported that PTOA was more common in patients with arthroplasty than ORIF (37% vs. 0%).

In the current study, only 22.7% (45/198) of patients were diagnosed with “MCL injury,” whereas Zhang et al. [28] reported an incidence of 71.4% (15/21) of MCL injury evaluated by magnetic resonance imaging (MRI). This difference is probably due to the various criteria. MRI can accurately detect partial tear of MCL, which is reported to heal in a way that does not affect prognosis after simple dislocations or fracture-dislocations of the elbow [29, 30]. In the report of Zhang et al., MCL injury was partially torn in 12 patients. And no one had a moderate or severe PTOA. We defined MCL injury as more than 3 mm of joint space widening by intraoperative fluoroscopy. It is used to find complete or large partial tears of MCL that can lead to instability of the medial side [31, 32]. Our result was in line with another study, in which Jung et al. [33] showed that only 17.1% of TTI patients were defined as MCL injury with a criterion of more than 5 mm of joint space widening, which denoted a complete rupture of MCL. Although MCL is a primary stabilizer for the elbow, little is known about the relationship between MCL injury and PTOA. Our results are in accordance with a previous study which indicated a high incidence of PTOA in the presence of an associated MCL injury [34].

All TTI leads to different degrees of instability resulting from the extent of the separate component injury of the elbow [35]. It has been reported that the complete rupture of MCL is related to recurrent instability of the elbow increasing the risk of PTOA [33]. We also found that patients with subluxation of the elbow were more likely to develop moderate or severe PTOA. Recurrent instability of the elbow can damage the articular cartilage. On the other hand, it results in the lesion of the repaired collateral ligaments which further increased the instability. However, the necessity of repairing MCL is still under debate. In a series of 16 TTI patients, Toros et al. [36] indicated no differences in functional score and incidence of PTOA between patients with and without surgical treatment for MCL. In contrast, Jeong et al. [37] recommended MCL repair and reported no moderate or severe PTOA in their study of 13 TTI patients. An MCL injury is often accompanied by avulsion of the muscles from the epicondyle resulting in increased instability [38]. Moreover, the repair of MCL is an effective procedure if a postoperative instability occurs. Hence, the repair of severe MCL injury seems to have a positive effect on the prevention of PTOA. But, it needs to be confirmed by further prospective studies.

Occupation is known to be a risk factor for the development of osteoarthritis on several joints, such as the hip, knee, ankle, and elbow [15, 39–41]. However, Lübbeke et al. [42] reported that heavy work did not increase the PTOA of the ankle. It is not surprising because the risk factors for primary osteoarthritis and PTOA may differ due to the different pathogenesis of them. For example, occupation-related osteoarthritis commonly occurs within the ulnohumeral joint, while PTOA shows specific forms resulting from different injury patterns [43]. Also, in a series of 139 patients with surgical treatment of different elbow injuries, Guitton et al. [44] indicated that injury type, but not heavy use of the elbow, was associated with PTOA. Interestingly, we found heavy use of elbow was also a significant risk factor for PTOA in the patients of the TTI. A possible explanation is that cartilage health is depended on the interaction of biological, mechanical, and structural components [45]. Compensation from biological and mechanical components may keep the joint in a state of asymptomatic prearthritis after structural damage of the elbow [45], whereas the TTI is more complex among all elbow injuries and is often caused by high energy injury, which can destroy the homeostasis of both biological and structural components. Moreover, the reaction force of the elbow is more than twenty times as the external load because of the short lever arm of the muscles [46]. Then, under these conditions, cartilage degradation may be aggravated by mechanical stimulation during heavy use of the elbow.

Identifying the risk factors for PTOA and quantifying their effects are important for both doctors and patients. It allows for the establishment of prognosis, which could be used to inform patients of the future risk of PTOA. Besides, viable treatment options can be adopted to prevent the undesired outcome because some risk factors might be modifiable or their impact reduced. In our study, the only potentially modifiable risk factor was occupation after fracture healing. Moreover, we also assessed the risk categories according to the number of risk factors. And our results showed that the incidence of moderate or severe PTOA was lower in patients without heavy use of the elbow than those with (Figure 1). It indicated that patients should be informed of this additional risk and counseled about avoiding heavy use of the elbow (such as changing another type of job or switching the hand during these works), especially for those with Mason III radial fractures.

With regard to the influence of other potential factors, several studies found a correlation between age [41], gender [41], overweight [47, 48], smoking [13, 49], alcohol consumption [13], diabetes mellitus [48], hypertension [48], and primary osteoarthritis. Contrarily, they did not influence the development of moderate or severe PTOA in the current study. It further confirmed the difference in risk factors between primary osteoarthritis and PTOA, whereas we found that alcohol abuse was significant in univariable regression analyses, as well as the type of coronoid fracture and surgical approach. None of them were significantly associated with the development of moderate or severe PTOA in multivariable logistic regression analyses. The lack of significance may be due to interaction among variables. For example, we found that alcohol abuse mostly occurred in patients who were heavy users of the elbow, combined approach denoting severe damage to medial structures. Besides, a displaced coronoid fracture often represents an avulsion of the anterior MCL [38]. We also found that severe coronoid fracture had a correlation with MCL injury and a more severe radial head fracture.

## 5. Limitations

Several limitations of this report should be explained. First, being a retrospective study, there were unavoidable losses of follow-up and ambiguous medical records. We had contacted some patients to confirm details, but recall bias influenced the accuracy of those data. Second, due to its relatively low incidence of the TTI, most single-center studies of the risk factors for complications had a small number of patients. It decreased statistical power. Therefore, we conducted a multicenter study. Additional cases also allowed us to analyze more potential factors. However, choices of fixation (such as implants for fractures and treatments of MCL injuries) were depended on surgeons' discretion. It may interfere with our results even though surgeries were performed based on standard strategy. Third, we only evaluated PTOA by radiographs alone. Limited relationships were reported among radiographic evidence of PTOA and symptoms or disability [50]. Moreover, some authors would prefer a longer-term follow-up to analyze PTOA. But cartilage degeneration related to age should be considered under this condition. It has been suggested that PTOA develops 2 to 6 years after initial injuries [22]. Therefore, our mean follow-up of 42.8 months should be adequate to capture differences in the early development of PTOA.

## 6. Conclusion

Moderate or severe PTO was common (32.3%) in TTI patients with surgical treatment. Risk increased with Mason III radial head fracture, MCL injury (medial instability), and heavy use of the elbow. The probability of developing moderate or severe PTOA is higher among patients with more risk factors. Patients should be counseled about avoiding heavy use of the elbow, especially for those with Mason III radial head fractures. In addition, surgeons should be aware of the recurrent instability. Further prospective studies are needed to confirm the effect of MCL treatment on the development of PTOA.

## Figures and Tables

**Table 1:** Patient demographics, injury, and treatment information.

	Mild or no PTOA (*n* = 134)	Moderate or severe PTOA (*n* = 64)	*p* value
Age, years, mean (±SD)	41.2 (±12.7)	42.1 (±11.5)	0.198
Gender, *n* (%)			0.806
Male	82 (61.2)	38 (59.4)	
Female	52 (38.8)	26 (40.6)	
Dominance, *n* (%)			0.822
Dominant	71 (53.0)	35 (54.7)	
Nondominant	63 (47.0)	29 (45.3)	
Diabetes mellitus, *n* (%)	17 (12.7)	5 (7.8)	0.307
Alcohol abuse, *n* (%)	45 (33.6)	31 (48.4)	0.044
Hypertension, *n* (%)	35 (26.1)	12 (18.8)	0.254
Smoking, *n* (%)	40 (29.9)	19 (29.7)	0.981
BMI			
Mean (±SD)	23.6 (±2.8)	24.4 (±2.9)	0.383
Overweight, *n* (%)	41 (30.6)	28 (43.8)	0.069
Occupation, *n* (%)			
Heavy use	28 (20.9)	24 (37.5)	0.013
Coronoid fracture, *n* (%)			<0.001
I	91 (67.9)	27 (42.2)	
II	36 (26.9)	22 (34.4)	
III	7 (5.2)	15 (23.4)	
Radial head fracture, *n* (%)			<0.001
I	10 (7.5)	1 (1.6)	
II	63 (47)	12 (18.6)	
III	61 (45.5)	51 (79.7)	
MCL injury, *n* (%)	17 (12.7)	28 (43.8)	<0.001
Time to surgery, days, mean (±SD)	4.9 (±3.5)	5.5 (±4.4)	0.311
Surgical approaches, *n* (%)			0.018
Lateral	95 (70.9)	34 (53.1)	
Posterior	12 (9.0)	5 (7.8)	
Lateral with medial	27 (20.1)	25 (39.7)	

(%) represents the percentage within each group.

**Table 2:** Univariate analysis: association between moderate or severe PTOA and patient demographics, injury, and treatment information.

	Total (*n*)	Moderate or severe PTOA	Odds ratio	95% confidence interval	*p* value
Age			1.006	0.982-1.031	0.624
Gender (%)					
Male	120	38 (31.7)	0.927	0.505-1.702	0.806
Dominance (%)	106	35 (33.0)	0.934	0.514-1.698	0.822
Diabetes mellitus (%)	22	5 (22.7)	0.583	0.205-1.659	0.312
Alcohol abuse (%)	76	31 (40.8)	1.858	1.012-3.410	0.046
Hypertension (%)	47	12 (25.5)	0.653	0.313-1.363	0.256
Smoking (%)	59	19 (32.2)	0.992	0.517-1.903	0.981
BMI (%)					
Overweight	69	28 (40.6)	1.764	0.953-3.265	0.071
Occupation (%)					
Heavy use	52	24 (46.2)	2.271	1.179-4.375	0.014
Coronoid fracture (%)					
I	118	27 (22.9)	Reference	Reference	Reference
II	58	22 (37.9)	2.060	1.041-4.076	0.038
III	22	15 (68.2)	7.222	2.671-19.528	<0.001
Radial head fracture (%)					
I/II	86	13 (15.1)	Reference	Reference	Reference
III	112	51(45.5)	4.695	2.337-9.430	<0.001
MCL injury (%)	45	28 (62.2)	5.353	2.634-10.877	<0.001
Time to surgery			1.045	0.968-1.127	0.258
Surgical approaches (%)					
Lateral	129	34 (26.4)	Reference	Reference	Reference
Posterior	17	5 (29.4)	1.164	0.382-3.548	0.789
Lateral with medial	52	25 (48.1)	2.587	1.323-5058	0.005

(%) represents the percentage of the total.

**Table 3:** Multivariate analysis: risk factors for moderate or severe PTOA.

	Odds ratio	95% confidence interval	*p* value
Alcohol abuse	1.445	0.716-2.914	0.304
Occupation (heavy use)	2.333	1.060-5.136	0.035
Coronoid fracture			
I	Reference	Reference	Reference
II	1.273	0.526-3.078	0.592
III	3.616	0.798-16.376	0.095
Radial head fracture			
I/II	Reference	Reference	Reference
III	4.049	1.877-8.736	<0.001
MCL injury	5.120	1.261-20.790	0.022
Surgical approaches			
Lateral	Reference	Reference	Reference
Posterior	0.599	0.162-2.221	0.443
Lateral with medial	0.319	0.070-1.460	0.141

**Table 4:** Incidence of moderate or severe PTOA according to the number of risk factors present.

Risk factors (*n*)	Patients (*n*) per risk factor category	Incidence of moderate or severe PTOA
0	59	5 (8.5%)^a^
1	81	25 (30.1%)^b^
2	46	24 (52.2%)^c^
3	12	10 (83.3%)^c^

Statistical significance *p* < 0.05 (^a^compared to 1, 2, and 3 risk factors; ^b^compared to 0, 2, and 3 risk factors; ^c^compared to 0 and 1 risk factors).

**Table 5:** Complications between the two groups.

Complications	Mild or no PTOA (*n* = 134)	Moderate or severe PTOA (*n* = 64)	*p* value
Heterotopic ossification	31	15	0.962
Subluxation	6	10	0.007
Neuropathy	13	4	0.417
Delayed union	8	3	0.712
Nonunion	2	2	0.445
Hardware migration	5	1	0.405
Infection	3	1	0.752

## Data Availability

The clinical data used to support the findings of this study are restricted by the Ethics Committee of our hospitals in order to protect patient privacy. Data are available from the corresponding author for researchers who meet the criteria for access to confidential data.
